# SIGLa: an adaptable LIMS for multiple laboratories

**DOI:** 10.1186/1471-2164-11-S5-S8

**Published:** 2010-12-22

**Authors:** Alexandre Melo, Alessandra Faria-Campos, Daiane Mariele DeLaat, Rodrigo Keller, Vinícius Abreu, Sérgio Campos

**Affiliations:** 1 Departamento de Ciência da Computacão, Instituto de Ciências Exatas Universidade Federal de Minas Gerais, Av. Antônio Carlos, 6627 - Pampulha, Belo Horizonte, MG, 31270-901, Brazil; 2 Centro de P & D em Recursos Genéticos, IAC, Campinas, SP, Brasil

## Abstract

**Background:**

The need to manage large amounts of data is a clear demand for laboratories nowadays. The use of Laboratory Information Management Systems (LIMS) to achieve this is growing each day. A LIMS is a complex computational system used to manage laboratory data with emphasis in quality assurance. Several LIMS are available currently. However, most of them have proprietary code and are commercialized with a high cost. Moreover, due to its complexity, LIMS are usually designed to comply with the needs of one kind of laboratory, making it very difficult to reuse a LIMS. In this work we describe the Sistema Integrado de Gerência de Laboratórios (SIGLa), an open source LIMS with a new approach designed to allow it to adapt its activities and processes to various types of laboratories.

**Results:**

SIGLa incorporates a workflow management system, making it possible to create and manage customized workflows. For each new laboratory a workflow is defined with its activities, rules and procedures. During the execution, for each workflow created, the values of attributes defined in a XPDL file (which describe the workflow) are stored in SIGLa’s database, allowing then to be managed and retrieved upon request. These characteristics increase system’s flexibility and extend its usability to include the needs of multiple types of laboratories. To construct the main functionalities of SIGLa a workflow of a proteomic laboratory was first defined. To validate the SIGLa capability of adapting to multiples laboratories, on this paper we study theprocess and the needs of a microarray laboratory and define its workflow. This workflow has been defined in a period of about two weeks, showing the efficiency and flexibility of the tool.

**Conclusions:**

Using SIGLa it has been possible to construct a microarray LIMS in a few days illustrating the flexibility and power of the method proposed. With SIGLa’s development we hope to contribute positively to the area of management of complex data in laboratory by managing its large amounts of data, guaranteeing the consistence of the data and increasing the laboratory productivity. We also hope to make possible to laboratories with little resources to afford a high level system for complex data management.

## Background

The advances in technologies present in biomedical research resulted in a large amount of data being generated by research, testing and commercial laboratories. With such large quantities of data, it becomes very difficult to control the quality of the processes and results generated. In order to address these issues the concept of a Laboratory Information Management Systems (LIMS) has been developed. LIMS are complex computational system used by a laboratory to manage its data. LIMS emphasizes quality assurance and aim to generate results in a consistent and trustworthy way [1]. They also manage the cycle of life of the data, that includes collection, storage, analysis and emission of reports. Several LIMS are available currently. However, most of them have proprietary code and are commercialized with a high cost, hindering its use by students and small laboratories. Moreover, the activities and procedures executed in the laboratories are, in general, quite different between distinct laboratories, making it difficult to build an adaptable LIMS. Therefore, due to its complexity, LIMS are usually designed to comply with the needs of only one kind of laboratory.

In this work we describe the Sistema Integrado de Gerência de Laboratórios (SIGLa), an open source LIMS with a new approach designed to allow it to adapt its activities and processes to various types of laboratory. To make SIGLa adaptable we use a workflow management system incorporated to the system. In SIGLa, for each new laboratory a new workflow will be defined with its activities, rules and procedures. After that, a file with the workflow’s definitions will be loaded in the system, in order to allow SIGLa to manage the activities of the laboratory. The definition of a new workflow is simple enough to be executed by a user knowing all the procedures of the laboratory, but without any prior programming knowledge.

The first workflow defined in SIGLA describes the activities of the proteomics’ laboratory of a UFMG Biochemistry laboratory [2]. This workflow has been defined in collaboration with proteomics specialists, in order to ensure that all laboratory requirements are reflected in the workflow. To validate the SIGLa capability of adapting to multiples laboratories, on this paper we study the process and the needs of a microarray laboratory and define its workflow. The first version of SIGLa can be accessed at http://luar.dcc.ufmg.br/sigla in the link SIGLa with the login guest and password guest. SIGLa has diverse functionalities that guarantee the quality of laboratory’s data and allow great flexibility in the construction of the workflow. With the development of SIGLa we hope to contribute positively to the area of management of complex laboratory data.

## Related work

Due to the complexity of LIMS, usually they are developed by large companies. As examples of commercial LIMS we have SQL LIMS [3], LabSoft LIMS [4] and LabWare LIMS [5], developed by private companies. Usually, these LIMS are specific for one type of laboratory. SQL LIMS, for example, has distinct solutions for pharmaceutical laboratories, chemical, nourishing, forensic and water analysis laboratories. The great diversity of laboratories is the reason for abundance of LIMS in the market. However, although companies try to adapt their systems for each customer, the task of finding the ideal LIMS for one specific laboratory is not easy. Even laboratories of the same type have particularities in the procedures that differentiate them. Unless it is a LIMS constructed specifically for the laboratory, an efficient use will frequently demand significant customization which is not always available or affordable. Some free open source LIMS are currently available. Generally they are limited, as the FreeLIMS, developed for the German company Labmatica. This company offers an open source version and a commercial version. The open source version, as is usually the case, has limitations when compared to the commercial one. Some free LIMS have been constructed as academic works. Some examples are [6], a LIMS developed for an academic microchip fabrication facility, with emphasis in the security of the LIMS; [7] a LIMS developed for cancer research laboratories and [8] and [9], LIMS developed for biological research laboratories. [8] developed a services based LIMS, with focus in the integration of the data stored in biological databases. The work [10] propose a LIMS to manage the maize mapping project data. Is important to notice that these are solutions for specific laboratories.

There exist some LIMS that also incorporate diverse concepts of workflows, such as [11] and [12] that manage data from laboratories of protein analysis. However they don’t have a workflow management system directly incorporated to the system, like SIGLa’s. The LIMS presented in [11] and [12] are specific for proteomic laboratories. In [12] it is stated that it can be adapted to other types of laboratories, however to do that it’s necessary to modify the system’s code. With SIGLa this adaptation can be made without any need to change the code, making it easier and more efficient to use.

## Tools for workflow development and management

A workflow can be defined as the steps and tasks executed sequentially according to a set of rules and procedures in order to conclude a process. A workflow can be a sequential progression of activities,or a complex set of processes occurring concurrently and eventually impacting in others, according to a set of rules [13]. A workflow management system allows defining and controlling the activities associated with the process. Usually a workflow management system has a tool for defining workflows. With this tool the activities, its attributes, the transitions between the activities and the rules for execution of the activities are defined. The workflow editor generates a file that contains the complete workflow and will be read by the workflow management system. The output file, containg the workflow definition, can follow some standards, like XPDL (XML Process Definition Language) [14], BPEL (Business Process Execution Language) [15] and BPML (Business Process Modelling Language) [16]. In this work we will use the XPDL standard, that currently is one of the most commonly used. The XPDL standard was created by the Workflow Management Coalition (WfMC) [13], a group created to promote and to keep workflows standards. A XPDL file is a XML file that follows the WfMC specifications and contains all the definitions of a specific workflow.

Currently there are many workflow management systems, commercial and open-source. Some examples of these are the Enhydra Shark [17], ObjectWeb [18] and wfmOpen [19]. These are open-source systems that use XPDL standard and have a proper workflow editor. On this work we use the Enhydra Shark engine, because it was the option with the best support and documentation, as well as the most amenable to be integrated in SIGLa. To create the workflow we use the free version of the workflow editor of Enhydra Shark, the Together Workflow Editor.

## SIGLa

SIGLa is a LIMS focused on the workflow of laboratory activities. It guides the user through the execution of each activity, informing the next activity/activities that can be executed. For each activity SIGLa stores its attributes as an eletronic notebook. In just one view the user can visualize all the activities executed in a experiment. The details of each activity can be accessed by just clicking on the activity. SIGLa adapts its interface to each laboratory as it is capable of managing workflows defined in the XPDL standard. This is possible because SIGLa uses a workflow management system to control which activities have been executed and which ones are available for execution.

It is an application with an easy to use interface that is easily adaptable to different types of laboratories, in contrast to most LIMS that support a single type of laboratory. To store and manage data on laboratory activities SIGLa uses workflows. In workflow based systems users define activities, transitions, actors and rules of transitions. In SIGLa it is possible (through the Together Workflow Editor) also to define further details for each entity. For example, during the laboratory workflow definition, the user can define the attributes of each activity, its types, the range of values that each attribute can assume, its formats or define auto-calculated attributes from other attributes. It’s also possible to define the inputs and outputs of each activity, to define the number of inputs and outputs, as well as the relation of these inputs and outputs with the experiments. In the workflow definition it is also possible to assign to each activity a documentation that contains standards, instruments calibration, procedures and registers associates to the activities.

It’s important to notice that to successfully manage a laboratory it’s necessary to create a well defined workflow. It must contain all the experiments with its attributes, inputs and outputs clearly declared with its types, formats and specified sizes. The definition of workflow is a very important step in the process of quality assurance given by SIGLa. Once the activities, rules and procedures have been defined, the workflow editor generates a XPDL file with all the definitions. This file is loaded in SIGLa, then the LIMS will be ready to manage the laboratory activities. With this mechanism, practically any type of laboratory can define its activities and rules in an XPDL file and use SIGLa to manage all the laboratory information. For SIGLa’s initial development the workflow of a proteomics laboratory was defined [2]. Proteomics is the process of identification and quantitative analysis of proteins expressed in different conditions or life stages of a cell or organism. Several analytical methods are used in proteomic analysis generating large amounts of data that varies significantly depending on the experiment type and conditions used. By using this kind of experiment as an model for SIGLA’s initial development we have shown how SIGLa’s can manage real complex experiment data.

After defining the proteomic workflow and implementing the main functionalities of SIGLa we have defined a second workflow to validate the capability of SIGLa of adapting to multiples laboratories. With the support of UFMG Microarray Laboratory we have defined the microarray workflow. The technique of DNA microarrays is used to study gene expression on a large scale in several species. DNA microarrays are usually layers of glass, plastic or nylon which is deposited series of thousands of microscopic spots of oligonucleotides or cDNAs, each containing picomoles of a specific DNA sequence. The microarray slide is then used to detected expression level of mRNA related to the DNA printed on its surface by incubatiing of the microarray with a solution containing cDNA or RNA obtained from biological samples. [20].

Microarray technique generates a large number of information, both laboratory data and image and data files. The microarray process has several steps, requiring the storage of information for each one of them. Usually this information is stored in lab books, which makes it difficult to access the information, since they are in chronological order only. After scanning microarray slides and image analysis, new and large image data and data files are produced and the number could reach hundreds of files. The organization of these data using laboratory notebooks or basic text files becomes very time-consuming. By using a single platform as SIGLa for this task is possible to keep all data organized making its manipulation more reliable. Moreover, the use of a web based platform such as SIGLas provides access to data for all members of a research group in a fast and efficient way.

The current version of SIGLa is able to manage all the activities of a workflow and their attributes. An important feature that is not currently available, however, is the ability to call an external program to perform an automatic analysis such as gene sequence annotation. In this case, the analysis has to be performed externally. Its result, however, can be stored in SIGLa as an attribute of type file, and can be used later in the workflow. Automatic analysis execution will be available in the next version of SIGLa.

## Microarray workflow

**Figure 1:**
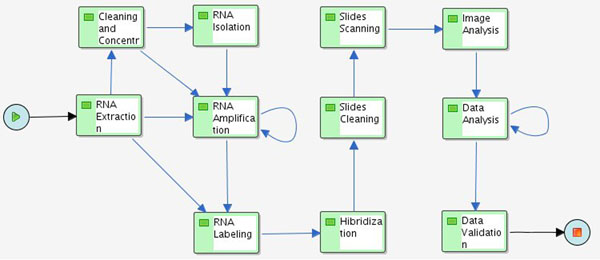
**MicroarrayWorkflow** as defined in SIGLa.

To implement the microarray process into SIGLa we have defined it’s workflow (figure 1). On this workflow the activities execution order is defined as well as a series of attributes. This guarantees that the activities will be executed in the correct order along with all associated information. For each attribute we define its type, that can be string, integer, date, real or file. We can set an attribute as *not null*, meaning that the value of the attribute must be always filled. It’s also possible to define the format of an attribute. The file type permits the association of files to an activity. For example, it’s possible o associate to the execution of the activity *Data Analysis* a file containing the annotation of the gene sequences in the microarray to be analyzed by another application. During the workflow definition we have also defined the inputs and outputs of the activities. Each activity generates outputs that will be inputs to the next activity. For example, in the case of RNA Extraction it generates the extracted RNA as output. In this case it is necessary to store the RNA total concentration, the RNA final volume, the Gel image and the dosage method. All these attributes are defined in the RNA Extraction activity, in the microarray workflow. After the RNA Extraction it’s possible to execute a Cleaning, an Amplification or a Labeling. In the workflow an input to these three activities was defined. In this way, the output of the RNA Extraction will be the input to the Cleaning, Amplification or Labeling activity. It’s also possible to set a property that can define the maximum and minimum number of each input and output of the activities.

### Workflow execution

**Figure 2:**
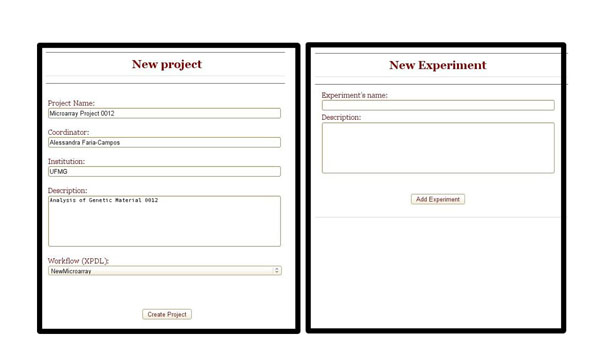
**New Project and New Experiment interface.** Interface to create a new Project and a new Experiment.

**Figure 3:**
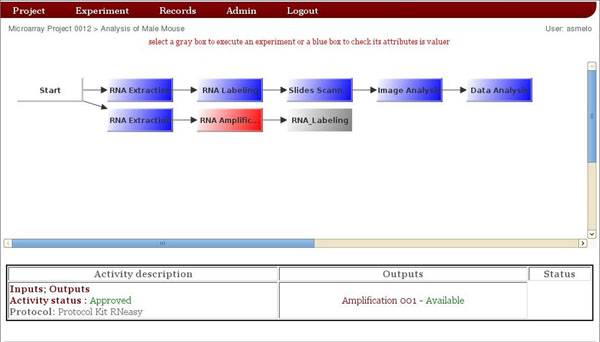
**Workflow Execution Interface.** Interface to manage workflow execution. Gray boxes are the activities available for execution. Blue boxes are the executed activities. The selected activity is represented by red boxes, and its attributes are shown in the table below the workflow.

To organize laboratory’s data, SIGLa has the following structure: A project has experiments where samples are analyzed. To begin a workflow execution the first step is to create a project and it’s associated experiments. Figure 2 shows the interface to create a project and the experiments. After the selection of a previously created experiment, SIGLa presents an integrated interface (Figure 3) where it’s possible to manage the complete workflow. Gray boxes are the activities available for execution, according with the workflow definition. Blue boxes are the executed activities. It’s possible to select an already executed activity to verify it’s attributes. The selected activity is represented by a red box, and its attributes are shown in the table below the workflow. In this screen you can manage multiple samples, and each row corresponds to the analysis of a sample. Figure 3 shows an example of the analysis of two samples, where each row corresponds to a sample.

**Figure 4:**
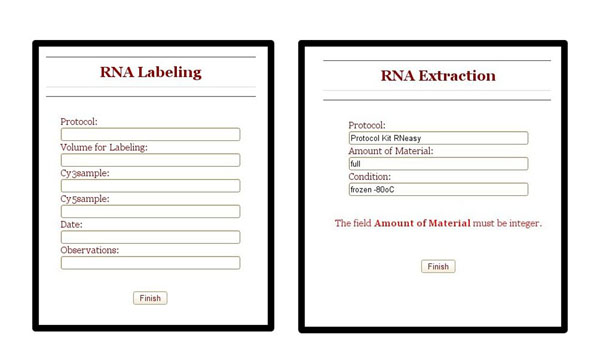
**Activity Execution interface.** Interface to fill the attributes of an activity. After filling the attributes SIGLa validates them, checking if the attributes were filled with the correct value type.

To execute an activity the user just needs to click in the correspondent gray box. Clicking on it, SIGLa will open an interface where the user can fill all the attributes that were defined in the workflow definition for this specific activity. When the user completes the activity, SIGLa checks the properties of each attribute defined in the workflow definition. If some attribute value is inconsistent, SIGLa presents an error message (Figure 4). This avoid the insertion of inconsistent data into the database and ensures quality. Allowed values to fill the activities attributes can also be pre-defined by the researcher and the values accessed through a dropdown list. Using this feature the user can’t insert a random value but just select the predefined one.

**Figure 5:**
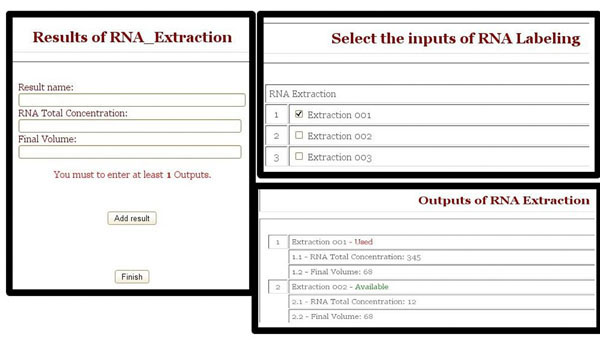
**Activity Output** Interface to define the results of an activity, to select its inputs and to consult its results.

SIGLa controls the selection of specific inputs and outputs for each experiment registered in the system. When the activity is defined to have outputs,after the execution of the activity, SIGLa opens a window on the interface for inserting the outputs. As in the activity execution, in the output insertion SIGLa checks the properties of each attribute and makes the validation, guaranteeing data quality. When a activity is defined to have inputs resulting from previous activities, before the execution of the activity SIGLa opens an window on the interface for selecting the input(s). On this screen SIGLa validates the number of inputs that have been selected. Once the activity inputs and outputs were defined for an activity the information about them can be accessed by the link inputs and outputs shown in the figure 3, in the Activity description table. Figure 5 shows the interfaces of outputs definition, inputs selection and consult of results. To help with the laboratory management, SIGLa offers a short report, listing all the executed activities in a certain experiment. On this report it’s possible to check when the activity was executed and who executed it. SIGLa also offers a long report that list all the executed activities of a experiment detailing the activities attribute values.

## Implementation

**Figure 6:**
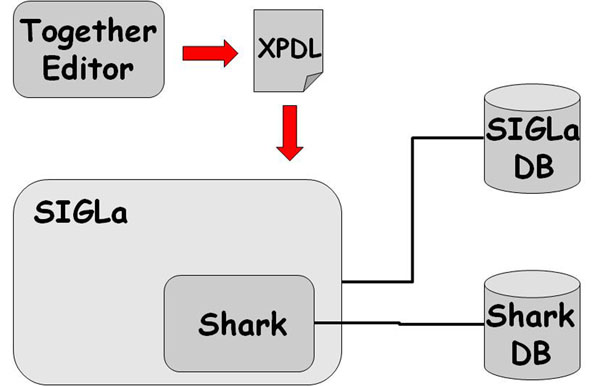
**SIGLa’s architecture** SIGLa’s architecturet

SIGLa uses JAVA as programming language and has a basic architeture (Figure 6). The Web pages are JSP and the J2EE technology is used for code development. Applets are used for the graphical interface. The database was developed with MySQL, a robust, scalable and free DBMS. To define a workflow,the Together Workflow Editor community edition was used with the XPDL standard. For managing the workflows SIGLa uses the workflow manager Enhydra Shark. A workflow manager consists of a set of functions that controls the activities of a workflow. The workflow manager maintains a list of activities that have been executed, as well as the order of execution and the activities that are available for execution. SIGLa calls Enhydra Shark functions whenever new activities are executed, and consequently workflow execution information is maitained inside SIGLa by Shark functions. There are many features of SIGLa that have been implemented as extensions of Shark. For example, Shark cannot directly allow an activity to be repeated. In a laboratory, frequently when a result is not clear enough, the experiment is repeated. Modelling this behavior in Shark is complex, and additional workflow management functions have been implemented to make this an efficient task. Another example is the concept of experiments inputs and results. As defined by Shark, a workflow is simply a sequence of events. In SIGLa, however, activities can generate results which can be used as inputs in other activities. This feature has been implemented in SIGLa because it does not exist in Shark.

## Results and discussion

Once the microarray workflow has been loaded into SIGLa, a specialist on microarray executed several experiments to validate the system. The system was tested with real data from a microarray experiment with 12 initial samples of RNA and six microarray slides. All steps of the workflow have been completed for all 12 samples. The system was robust and easy to use during the test. The ability to see in one screen which parts have been executed and which ones have not made the process much simpler. After completing all the steps, the specialist could verify its data for any stage. This access to all information of an experiment is very useful when working with a technique complex and full of steps as microarray analysis. In addition, the system stores protocols and files generated during the execution of steps, such as pictures of gels or data files, making it a good tool for storing data in an experimental laboratory for the implementation and analysis of microarrays. SIGLa helped also with other important features such as allowing a choice between two or more possible options in a field, as the choice of fluorescent dyes Cy3 and Cy5, which facilitates the work of researchers when they are filling these fields. Moreover, it was possible to generate reports in PDF format with all the information of the experiment (Long and Short Report), which can be easily viewed and printed. The attribute values validation made by SIGLa was also very useful. In many moments it avoided filling the fields with wrong values. In addition, testing with real data allowed the specialist to make suggestions to improve the system, which will be analyzed and insert in the original design.

It is important to notice that the period of time to fully create a complete microarray LIMS has been about two weeks. This illustrates that SIGLa is able to adapt itself to multiple laboratories in very little time. The process consists of defining the workflow in a graphical editor, and SIGLa automatically creates all the data structures needed to manage the laboratory. Any modification in the protocols, or the addition of new experiments takes only a few days making it possible to manage not only large quantities of data, but also different types of data efficiently.

## Conclusion

The need for fast and reliable data storage and management for biological laboratories is a reality nowadays. This need has been fulfillled only partially by available sistems given high costs or limitations of the available LIMS. In this work we present SIGLa, a system based on adaptable workflows, with an easy to use interface, that manages and guarantees the quality and integrity of laboratory data. Moreover SIGLa is not a solution designed for only one type of laboratory but several types, since the user can easily adapt it to the needs of his/her laboratory, simply by defining its workflow. On this paper we study the process and the needs of a microarray laboratory and define its workflow. This workflow has been used by researchers in real microarray experiments and they have reported that the system indeed has been very useful. With SIGLa’s development we hope to contribute positively to the area of management of complex data in laboratory by managing its large amounts of data, guaranteeing the consistence of the data and increasing the laboratory productivity. We also hope to make possible to laboratories with little resources to afford a high level system for complex data management.

## Availability and requirements

• **Project name:** Sistema Integrado de Gerenciamento de Laboratórios (SIGLa)

• **Project home page: http://www.luar.dcc.ufmg.br/sigla**

• **Operating system(s):** Platform independent

• **Programming language:** Java

• **Other requirements:** Java 1.5.0 or higher

• **Any restrictions to use by non-academics:** licence needed

## Competing interests

The authors declare that they have no competing interests.

## Authors’ contributions

On this work the decisions of implementation were made by AM, AFC and SC. AM, RK and VA wrote the java code. DDL and AFC gave the biological concepts for the implementation.
